# EHMTI-0314. A window to the past: the association between inflated pain scores and a history of abuse in women with chronic migraine

**DOI:** 10.1186/1129-2377-15-S1-D66

**Published:** 2014-09-18

**Authors:** BB Vargas, RB Halker, AJ Starling, AL Green, JG Hentz

**Affiliations:** 1 Neurology, Mayo Clinic, Phoenix, USA

## Introduction

Childhood abuse is a risk factor for depression and anxiety and is reported by 58% of patients with episodic migraine (EM) or chronic migraine (CM). Maximum headache severity >10/10 on a 10-point visual analog scale (VAS) is not uncommon among CM patients. The relationship between inflated pain scores and abuse has never been investigated.

## Aims

To evaluate the relationship between pain severity >10/10 on a ten-point VAS and a history of abuse in women with CM.

## Methods

A retrospective review was conducted on new headache consults in female patients with ICHD-IIIβ-defined CM over a 34-month period. Age, maximum headache severity on a 10-point VAS, and history of physical, sexual, and emotional abuse were collected.

## Results

80 patients were included. Average age was 43 years in the pain >10 group and 40 years in the pain ≤10/10 group (range 19-72 years; 95% CI -3 to 10, p=0.31). 28.8% (n=23) reported maximal pain >10/10 and 71.2% (n=57) reported maximum pain ≤10/10. Of those reporting pain >10/10, 87% (n=20) reported an abuse history compared to 35% (n=20) in the pain ≤10/10 group (OR 12, 95% CI 0.33 to 0.70, p<0.001).

## Conclusions

Among female CM patients, pain >10/10 correlates with a markedly higher likelihood of abuse compared to those reporting pain ≤10/10. As history of abuse is a risk factor for depression and anxiety, and treatment of psychopathology can influence CM treatment outcomes, all CM patients should be screened for a history of abuse, with special attention to those reporting pain >10/10.

No conflict of interest.

